# Psychological inflexibility explains social anxiety over time: a mediation analyses with a clinical adolescent sample

**DOI:** 10.1007/s12144-023-04650-w

**Published:** 2023-04-20

**Authors:** Diana Vieira Figueiredo, Francisca Alves, Paula Vagos

**Affiliations:** 1 Center for Research in Neuropsychology and Cognitive Behavioral Intervention – CINEICC, Faculty of Psychology and Educational Sciences, University of Coimbra, Coimbra, 3000-115 Portugal; 2 Institute of Human Development, Portucalense Infante D. Henrique University, Porto, 4200-072 Portugal; 3 William James Research Center, Departamento de Educação e Psicologia, Universidade de Aveiro, Aveiro, Portugal

**Keywords:** Social anxiety disorder, Adolescents, Psychological inflexibility, Longitudinal mediation

## Abstract

Social Anxiety Disorder (SAD) has its usual onset during adolescence when it is a highly prevalent and debilitating condition. Evidence regarding the processes that underline social anxiety and SAD is not compelling, especially in adolescents. Within an Acceptance and Commitment Therapy (ACT) framework, the causal role of ACT processes on adolescents’ social anxiety and how these processes contribute to sustain social anxiety over time is still unknown. Hence, this study explored the role of psychological inflexibility (PI) and acceptance and committed action (as psychological flexibility processes) on social anxiety over time, in a clinical sample of adolescents. Twenty-one adolescents (Mage = 16.19, SD = 0.750) with a primary diagnosis of SAD completed a set of self-report measures assessing PI, acceptance (i.e., willingness to experience social anxiety symptoms), action (i.e., moving towards valued life directions despite social anxiety symptoms) and social anxiety. Path analysis was used to investigate a mediation model linking acceptance, committed action, and PI to social anxiety, directly and indirectly. Findings revealed that acceptance and action were negatively and directly associated with PI after 10-weeks. In turn, PI yielded a positive and direct effect on social anxiety after another 12-weeks. PI totally mediated the relation between acceptance and action and social anxiety, with significant indirect effects. Overall, findings offer evidence for the applicability of the ACT model to adolescent SAD and support the use of clinical interventions targeting PI to understand and alleviate adolescents’ social anxiety.

According to the World Health Organization (WHO, 2021) about 14% of adolescents experience mental health problems. Social Anxiety Disorder (SAD) is the third most common disorder in adolescence (Kessler et al., 2005), with estimated prevalence rates during this life stage varying considerably - from 1.29% (Jystad et al., 2021) to 9.1% (Merikangas et al., 2010). It is characterized by a marked and persistent fear in social and/or performance situations in which one may be exposed to the scrutiny of others (American Psychiatric Association, 2013). Difficulties associated with SAD tend to present a persistent course with rare spontaneous remissions (Beesdo-Baum et al., 2012).

The onset of SAD usually occurs during adolescence (Stein et al., 2017; Knappe et al., 2015; Kessler et al., 2005) and adolescents with this disorder often experience significant negative impairments in several areas of life such as romantic relationships (Hebert et al., 2012), friendships (Acquah et al., 2015; Chiu et al., 2021), and academic performance (Soohinda & Sampath, 2016). Besides, research suggests that social anxiety is often comorbid with other anxiety and mood disorders (Jystad et al., 2021; Mohammadi et al., 2020), behavioral disorders (Mohammadi et al., 2020), and substance abuse (Jystad et al., 2021). The negative costs to adolescents’ life related to SAD, as well as its high comorbidity with other mental health difficulties, denote the relevance of continuing to examine the processes associated with this disorder, especially from a transdiagnostic point of view. Acceptance and Commitment Therapy (ACT) offers such a transdiagnostic perspective.

## Psychological (in)flexibility

ACT theorizes that Psychological Inflexibility (PI) contributes to maintain and exacerbate psychological disorders and human suffering (Hayes & Strosahl, 2005; Hayes et al., 2011). Accordingly, PI has been associated with multiple mental health disorders (Levin et al., 2014). From an ACT perspective, PI refers to rigid attempts to control, alter or minimize unpleasant internal experiences, which results in an inability to change and/or persist in value-guided behaviors (Hayes et al., 2006). PI stems from six interrelated processes, namely cognitive fusion, experiential avoidance, attachment to the conceptualized self, dominance of the conceptualized past/feared future, lack of values clarity and inaction, impulsivity, or avoidant persistence (Hayes et al., 2011).

Alternatively, Psychological Flexibility (PF) refers to the ability to be in contact with the present moment regardless of unpleasant internal experiences while altering or persisting in behaviors that are consistent with valued life directions (Hayes et al., 2006). In another words, it involves willingness to experience and embrace inner experiences without unnecessary attempts to change their frequency or form – acceptance – and the ability to move towards chosen values and goals despite unpleasant internal experiences – committed action. PF stems from six interrelated processes that are opposed to the PI processes, specifically acceptance, cognitive defusion, contact with present moment, self as context, values, and committed action (Hayes et al., 2006). Interventions focused on promoting PF have been associated with flourishing (Bohlmeijer et al., 2015) and wellbeing (Wersebe et al., 2018; Räsänen et al., 2016).

### Psychological (in)flexibility and mental health

Evidence for the association between PI/PF and its related processes with adults’ mental health difficulties has been gathered. In non-clinical samples, PF has been negatively associated, for example, with symptoms of depression (Makriyianis et al., 2019; McCracken et al., 2021), insomnia (McCracken et al., 2021), high levels of public speaking anxiety (Gallego et al., 2020) and anxiety (Makriyianis et al., 2019; McCracken et al., 2021). The alternative, PI, has been positively associated with measures of depression, anxiety (Bardeen & Fergus, 2016; Makriyianis et al., 2019) and posttraumatic stress (Bardeen & Fergus, 2016) and negatively associated with PF (Makriyianis et al., 2019). PI was also found to relate to a range of both current and lifetime depressive and anxiety disorders in a clinical sample of young adults (17–20 years old; Levin et al., 2014).

Concerning the specific processes relating to PI/PF, cognitive fusion has been positively to mediate the relationship between psychological well-being and psychopathological symptomatology in adults (Faustino et al., 2021) in non-clinical samples of adults. When comparing clinical and non-clinical adult samples, findings provide support for higher levels of cognitive fusion in the clinical sample (Faustino, 2021). Furthermore, a bidirectional relationship between cognitive fusion and experiential avoidance was found in a clinical sample of adults – this interaction accounted for a significant proportion of the association between stressful life events and anxiety and depression (Cookson et al., 2019). In Makriyianis et al. (2019) study with a non-clinical sample of colleges students, anxiety was negatively correlated to self as context, defusion, and committed action; alternatively, anxiety correlated positively with lack of present moment awareness, self as content, lack of contact with values, fusion, inaction, and experiential avoidance. Cognitive fusion was also found to relate to emotional regulation difficulties independently of diagnosis (Faustino, 2021).

Still, research on the role of PI/PF and its associated processes on adolescents suffering and well-being is not as compelling. Cobos-Sánchez et al. (2020) found an association between difficulties regulating emotions and PI, in a nonclinical sample of adolescents. Findings from a longitudinal study with a non-clinical sample of adolescents suggested that acceptance plays a causal role in adolescents well-being. Specifically, acceptance predicted increased positive affect and decreased sadness and fear after a 1-year period (Ciarrochi et al., 2010). PI, cognitive fusion, and experiential avoidance positively correlated with problems in controlling impulsivity, understanding emotions, achieving goals in distressing situations, non-acceptance of negative emotions and limited emotion regulation strategies. In line with these findings, Oppo et al. (2019) found that PI accurately predicted 78% of cases with self-reported internalizing problems in a sample of adolescents. Among participants who reported higher levels of depression/anxiety, the percentage of adolescents with high PI was around 90% (Oppo et al., 2019).

### Psychological (in)flexibility and social anxiety

Though some research has considered internalizing difficulties overall (Landy et al., 2015; Twohig & Levin, 2017), little is known about the specific ACT processes underlying social anxiety, particularly at its psychopathological levels, and using adolescent samples. Concerning non-adolescent samples, Tillfors et al. (2015) found a strong positive relationship between social fear/anxiety, social avoidance, and PI in a non-clinical adult sample. Additionally, in a college sample, the acceptance of socially anxious thoughts and feelings was found to be negatively associated with fears of being scrutinized and social interaction anxiety (Flynn et al., 2019). In a study with adults diagnosed with anxiety disorders (including SAD) PI was associated and significantly predicted depression, social anxiety, nonspecific anxiety, and panic measures (Fergus et al., 2012). Asher et al. (2021) observed that changes in experiential avoidance and social anxiety reciprocally mediated changes in each other in a sample of adults with SAD. Moreover, in this study, changes in experiential avoidance explained approximately 89% of changes in social anxiety.

There is further evidence in adult population that targeting PI is beneficial for several disorders (for a review see Gloster et al., 2020), including SAD (e.g., Khoramnia et al., 2020; Yadegari et al., 2014; for reviews see Caletti et al., 2022 or García-Perez & Valdivia-Salas, 2018), though the results have yet to demonstrate ACT as overall outperforming Cognitive Behavior Therapy (e.g., Herbert et al., 2018; Kocovski et al., 2013; Yabandeh et al., 2019). The components of ACT have been found to mediate change in the treatment of multiple clinical conditions (Forman et al., 2012), including the finding that acceptance brought about changes in social anxiety as reported by adults receiving a self-help book-based intervention (Kocovski et al., 2019).

Regarding adolescents’ social anxiety difficulties, existing research with non-clinical samples has also demonstrated that experiential avoidance is positively correlated with social anxiety (Kashdan et al., 2014; Papachristou et al., 2018; Shimoda et al., 2018), as well as cognitive fusion (Cheng et al., 2021). Moreover, results from a sample of inpatient adolescents (including adolescents with SAD) showed a significant relationship between anxiety disorders and experiential avoidance (Venta et al., 2012). Some studies have shown that ACT applied to adolescents with SAD is effective in treating this disorder in comparison to a control group (Oyetunde & Ajibola, 2019) and that ACT may play an important role in reducing social anxiety and in increasing distress tolerance and emotional regulation in adolescents (Roohi et al., 2019).

Despite the growing evidence of ACT processes underlying adolescent’s mental health problems in general and adolescent’s SAD in particular, evidence for the applicability of this model to explain SAD is scarce. Specifically, to our knowledge, no study has explored specific paths linking acceptance, committed action, PI and social anxiety in clinical samples of adolescents. Besides, few studies used longitudinal designs making conclusions about the causal role of PI processes on adolescents’ social anxiety limited.

### Purpose of the current study

The present study aimed to address those issues by examining the relationship between acceptance, committed action, and PI, and how those processes contribute to sustain social anxiety over time in a sample of adolescents with SAD. A longitudinal research design with three measurement time points was used. Specifically, a mediation model across time was tested, wherein acceptance and action in the first assessment moment was placed to predict PI in the second assessment moment (10 weeks later) and social anxiety in the third assessment moment (another 12 weeks later); PI was also placed to predict social anxiety in the third assessment moment.

We expected that acceptance and action would yield significant negative direct effects on social anxiety and PI. This is in line with evidence from non-clinical adult samples in which PF and its underlying processes have been negatively associated with anxiety (e.g., McCracken et al., 2021) and PI (e.g., Makriyianis et al., 2019). Alternatively, we expected PI to yield a significant positive direct effect on social anxiety. This is in accordance with previous findings regarding the positive associations between PI and social anxiety in non-clinical samples of adolescents (Papachristou et al., 2018; Shimoda et al., 2018) and in clinical samples of adults (Asher et al., 2021; Fergus et al., 2012). We also hypothesize that PI would partially mediate the relationship between acceptance and action and social anxiety. From an ACT perspective we expected that part of the effect of low levels of acceptance and action on social anxiety would be due to these processes contributing to an overall inflexibility and rigidity, in another words, contributing to PI. Lastly, we expected acceptance, action, and PI to account for a significant percentage of the variance of social anxiety.

## Method

This work refers to data collected within the procedures defined for the research project TeenSAD: Changing the Course of Social Anxiety in Adolescence (ClinicalTrials.gov Identifier: NCT04979676).

### Participants

The sample consisted of 21 adolescents with a primary diagnosis of SAD aged between 15 and 18 years old (M = 16.19, SD = 0.750). Adolescents were attending the 10th (*n* = 8; 38.1%) and the 11th (*n* = 13; 61.9) grades. Sixty-two (61.9%) percent of the adolescents were female (*n* = 13) and 38.1% were male (*n* = 8). Girls (M = 16.00, SD = 0.707) and boys (M = 16.50; SD = 0.756) had similar mean ages (t_(19)_ = -1.534, *p* = .142). According to parents’ occupation^1^, 57.1% of the participants came from low socioeconomic status (SES) households (*n* = 12), 38.1% from medium SES households (*n* = 8), and 4.8% from high SES households (*n* = 1). Boys and girls were homogeneously distributed by SES (χ^2^_(2)_ = 0.681, *p* = .711). Twelve participants (57.1%) presented only one diagnosis (SAD), eight (38.1%) presented one additional diagnosis (Panic Disorder [*n* = 3], Specific Phobia [*n* = 2], Attention-Deficit Disorder [*n* = 2] and Agoraphobia [*n* = 1]) and one presented five additional diagnoses (Panic Disorder, Agoraphobia, Major Depressive Episode single episode, Posttraumatic Stress Disorder and Obsessive-Compulsive Disorder). No differences were found between boys and girls regarding number of diagnoses presented (t_(19)_ = 1.658, *p* = .114). Adolescents were not receiving any psychological treatment during the three assessment moments.

### Procedures

The Study’s procedures were approved by the ethics committee of the hosting institution (January 15th, 2018) before any contact with schools and participants took place. The sample was collected from students attending the 10th or 11th grades from six Portuguese high schools, after consent from executive boards of the schools was granted. Written and informed consent from the guardian or legal representative of all students under the legal age of consent in Portugal (i.e., 18 years old) was requested. Potential participants were also informed on the goals and procedures of the research and the confidentiality and anonymity of their responses were guaranteed. Informed assent was requested from all adolescents. They were then asked to voluntarily participate in the study.

First, they were asked to fill in the Portuguese version of the Social Anxiety Scale for Adolescents (SAS-A; Cunha et al., 2004). Those who scored more than one standard deviation above the mean observed in a large normative Portuguese sample on the SAS-A (Cunha et al., 2004) were invited for an individual clinical interview (MINI-KID, Sheehan et al., 2010; Portuguese Authorized version by Rijo et al., 2016). The interview allowed the assessment of specific inclusion criteria, namely: (1) being aged between 15 and 18 years old; (2) having a primary diagnosis of SAD (DSM-5; American Psychological Association, 2013); and (3) having no indication of educational specific needs or psychotic symptoms. Adolescents meeting inclusion criteria were asked to complete a set of self-report questionnaires at three different moments, namely T0, then T1 that happened 10 weeks after T0, and T2 that happened 12 weeks after T1. Again, informed consent was obtained from parents or legal guardians and verbal assent was obtained from adolescents themselves to take part in the three assessment moments. The research protocol was sent via e-mail to the adolescents to be answered at home in an online survey platform (i.e., Limesurvey) and took approximately 30 min to answer.

### Instruments

All instruments were used in their Portuguese version.

#### Participants selection

##### Social Anxiety Scale for Adolescents (SAS-A; La Greca & Lopez, 1998; Portuguese version by Cunha et al., 2004)

The SAS-A is a self-report questionnaire comprised of 22 items (e.g., “I worry that others don’t like me”) that assess adolescents’ social anxiety experiences. Participants indicate the extent to which each item is “is true for you” on a 5-point Likert-scale (1 = ‘*not at all*’ to 5 = ‘*all the time*’). Besides the total score, the scale comprises three other subscales - the Fear of Negative Evaluation (FNE), the Social Avoidance and Distress of New Situations (SAD-New), and the Generalized Social Avoidance and Distress (SAD-General).

In the current work, only the total score was used for initial screening of participants. It had achieved a good internal consistency value for the Portuguese version of the instrument (i.e., α = 0.88; Cunha et al., 2004), and using the current sample (i.e., α = 0.85).

##### Mini International Neuropsychiatric Interview for Children and Adolescents (Mini-KID; Sheehan et al., 2010; Portuguese version by Rijo et al., 2016)

The Mini-KID is a structured clinical diagnostic interview which assesses DSM-V Axis I diagnoses in children and adolescents. It is organized in diagnostic sections, each presenting screening questions for each disorder. If positively answered, additional symptom questions are available for the evaluation of specific diagnostic criteria and diagnostic establishment. All questions are presented in a yes/no format. In its original version, interrater reliability was excellent across diagnoses except for dysthymia (Sheehan et al., 2010).

The Portuguese version of Mini-KID resulted from a thorough translation and backtranslation process and has been previously used as a method for diagnoses (Rijo et al., 2016). Clinicians applying the Mini-KID went through specific training, including role-play exercises and observation of experienced evaluators using the interview, before conducting interviews independently.

#### Data collection protocol

##### Social Anxiety and Avoidance Scale for Adolescents (SAASA; original version by Cunha et al., 2008)

The SAASA is a self-report questionnaire that assesses the degree of anxiety and frequency of avoidance in social situations representative of the most frequent social fears during adolescence. In the current work, its adapted version for late adolescents (Vagos et al., 2013) was used. It consists of 30 items (e.g., “Going to a party given by a colleague”) rated both for an anxiety and avoidance subscale on a five-point Likert scale (ranging from 1 = ‘*none*’ to 5 = ‘*very much*’ for anxiety; and from 1 = ‘*never*’ to 5 = ‘*almost always*’ for avoidance). Each subscale is comprised of six factors: interaction with the opposite sex, assertive interaction, observation by others, interaction in new social situations, performance in social situations, and eating and drinking in public.

In its original Portuguese version, the SAASA presented good internal consistency, with Cronbach’s alpha values over 0.87 for factors and total scores (Cunha et al., 2008). In its adapted version for older adolescents, factors attained adequate internal consistency values with Cronbach’s alphas over 0.70 (Vagos et al., 2013). The SAASA has also demonstrated good convergent and divergent validities as well as test-retest reliability (Cunha et al., 2008). Additionally, the SAASA has shown sensitivity to treatment results (Salvador, 2009) and the capacity to discriminate adolescents with SAD from adolescents with other anxiety disorders and without psychopathology (Cunha et al., 2008).

In the present study, only the total score for the anxiety subscale was used. It achieved excellent internal consistency values, with Cronbach’s alphas of 0.95 at T0, 0.94 at T1 and 0.95 at T2.

##### Social Anxiety–Acceptance and Action Questionnaire (SA-AAQ; MacKenzie & Kocovski, 2010; Portuguese adolescent version – SA-AAQ-A – by Martins et al., 2011)

The SA-AAQ-A is a self-report measure that consists of 19 items (e.g., “Being anxious in social situations makes it difficult for me to live my life as I would like to”) rated in a 7-point Likert scale (ranging from 1 = ‘*never true*’ to 7 = ‘*always true*’). It assesses experiential acceptance associated with social anxiety symptoms. In its original adult version, the SA-AAQ revealed a unidimensional structure and presented an excellent internal consistency (i.e., α = 0.94). It has also demonstrated positive correlations with other mindfulness and acceptance related measures, and negative correlations with social anxiety measures, depressive symptoms and thought suppression (MacKenzie & Kocovski, 2010).

In its Portuguese version for adolescents, the SA-AAQ-A revealed a two-factor measurement model: (1) Acceptance - Willingness to experience social anxiety symptoms; and (2) Action - Moving towards valued life directions despite social anxiety symptoms. The Acceptance and Action subscales revealed excellent (α = 0.92) and acceptable (α = 0.78) internal consistencies, respectively. Evidence for moderate temporal stability, construct validity in relation to measures of social anxiety, mindfulness/acceptance, and other anxious and depressive symptoms, and criterion validity in relation to the presence/ absence of SAD was also found for the SA-AAQ-A Portuguese version (Martins et al., 2011).

Using the current sample, the Acceptance subscale achieved good to excellent internal consistency with Cronbach’s alphas of 0.87 at T0, 0.94 at T1 and 0.94 at T2. The Action subscale presented adequate to good internal consistency, with Cronbach’s alphas of 0.72 at T0, 0.73 at T1 and 0.87 at T2.

##### Avoidance and Fusion Questionnaire for Youth (AFQ-Y; Greco et al., 2008; Portuguese version by Cunha & Santos, 2011)

The AFQ-Y is a 17-item self-report questionnaire that assesses psychological inflexibility, based on cognitive fusion and experimental avoidance. Items (e.g., “My life won’t be good until I feel happy”) are rated on a 5 point-Likert scale (ranging from 0 = ‘*Not at all True*’ to 4= ‘*Very True*’). In its original version, the AFQ-Y presented a unidimensional structure and a good internal consistency (α = 0.90). It also correlated positively with measures of anxiety, somatic complaints, problem behavior and thought suppression; it correlated negatively with measures of overall quality of life, acceptance, and mindfulness (Greco et al., 2008).

The Portuguese version of the AFQ-Y also achieved a unidimensional structure. It demonstrated a good internal consistency, with a Cronbach’s alpha value of 0.82 and a moderate temporal stability. Evidence was found regarding AFQ-Y construct validity in relation to measures of acceptance/ mindfulness, depressive and anxious symptoms, and social comparison (Cunha & Santos, 2011).

In our sample, the AFQ-Y achieved good internal consistency values, with Cronbach’s alphas of 0.89 at T0, 0.87 at T1 and 0.88 at T2.

### Data analyses

Initial statistical analyses were conducted using IBM SPSS Statistic 22 (Statistical Package for the Social Sciences version 22; IBM Corp.). No missing values were found for any variable on the assessment moments that were used in the current path analysis (see below). Data was found to follow a multivariate normal distribution based on the Mardia’s test of multinormality (Skewness = 9.390, *p* = .978; Kurtosis=-1.392, *p* = .164). The Maximum Likelihood Robust estimator was used. No multivariate outliers were found through Mahalanobis distance (p > .001). To detect multicollinearity, the variance inflation factor (VIF < 5) and the correlation matrix for all constructs were examined (Kline, 2005).

**Fig. 1:**
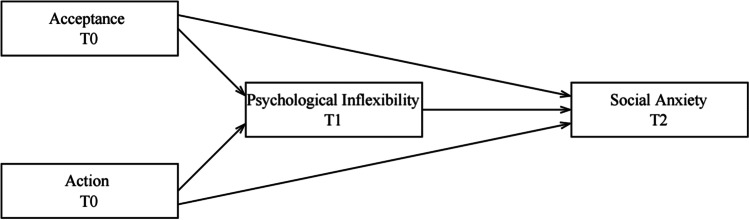
Baseline model. *Note.* Acceptance and Action measured by SA-AAQ-A acceptance and action subscales, respectively, at T0. Psychological Inflexibility measured by AFQ-Y at T1. Social Anxiety measured by SAASA at T2

Path analysis was conducted using Mplus v7.0 software (Muthén & Muthén, 2010). Social Anxiety measured at T2 was entered as the dependent variable and Acceptance and Action measured at T0 were entered as independent variables. Indirect effects between the independent and dependent variables were also considered through Psychological Inflexibility measured at T1. A model generation approach was followed, in which an a priori model was tested on the data (Fig. 1), and it was sequentially improved with only one modification at a time, based on theoretical considerations and statistical indications, until acceptable fit values were achieved. Acceptable fit considered a two-index strategy, based on Standardized Root Mean Square Residual (SMRM) < 0.09 combined with Comparative Fit Index (CFI) > 0.95 (Hu & Bentler, 1999). Additionally, through path analysis, the percentage of variance of Social Anxiety and Psychological Inflexibility explained by the respective predictive variables were analysed.

## Results

### Preliminary analyses

Means and standard deviations of all study variables for the total sample and for boys and girls separately are presented in Table 1. No differences were found between boys and girls nor between diverse SES levels, for any of those variables. Correlations between age and variables under study revealed non-significant associations between them.

**Table 1:** Means, standard deviation and matrix of inter-correlations among study variables

	(1)	(2)	(3)	(4)	*M (SD)*		
					Total sample (*N* = 21)	Girls (*n* = 13)	Boys (*n* = 8)
(1) Action	-				20.048 (5.417)	19.462 (5.897)	21.000 (4.751)
(2) Acceptance	0.478*	-			39.810 (12.910)	39.539 (14.158)	40.250 (11.498)
(3) Psychological Inflexibility	− 0.701**	− 0.619**	-		44.762 (10.478)	46.462 (10.325)	42.000 (10.810)
(4) Social Anxiety	− 0.682**	− 0.651**	0.801**	-	85.857 (23.327)	85.615 (24.965)	86.250 (22.044)
(5) Age	− 0.396	0.019	0.051	0.085	16.190 (0.750)	16.00 (0.707)	16.5 (0.756)

Acceptance and Action measured by SA-AAQ-A acceptance and action subscales, respectively, at T0. Psychological Inflexibility measured by AFQ-Y at T1. Social Anxiety measured by SAASA at T2. *M* = Mean; *SD* = Standard Deviation; *p < .05; **p < .001

Social anxiety associated significantly with all other variables. Specifically, higher levels of social anxiety associated with higher levels of PI; the reverse was true for the associations between social anxiety and the acceptance and action measures. Action and acceptance correlated positively with each other and negatively with PI. Despite the moderate to high correlations between study variables (cf. Table 1), the variance inflation factor indicates no multicollinearity problems were found (VIF < 5).

### Path analyses

**Fig. 2:**
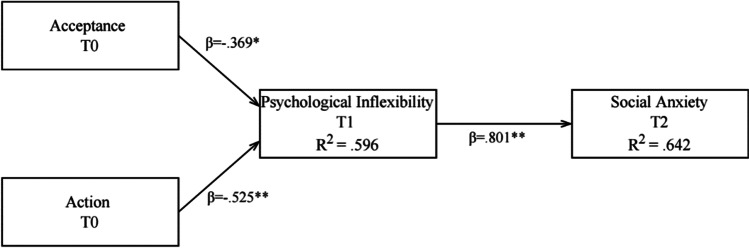
Final model. *Note.* Acceptance and Action measured by SA-AAQ-A acceptance and action subscales, respectively, at T0. Psychological Inflexibility measured by AFQ-Y at T1. Social Anxiety measured by SAASA at T2. *p < .05; **p < .001

Concerning path analysis, the baseline model (cf. Figure 1) was just identified (cf. Table 2). Changes to this model were then made based on the following criteria: (1) exclusion of all non-significant pathways and (2) inclusion of theoretically relevant pathways suggested by the modification indices. Specifically, the non-significant paths linking action and acceptance to social anxiety were excluded one at a time, and acceptable model fit indices were achieved (cf. Table 2). Criterion 2 was for model improvement not applied. The final model and the variance explained by this model are depicted in Fig. 2.

**Table 2:** Fit indices for competing models on acceptance, action, and psychological inflexibility as predictors of social anxiety

	χ^2^	*df*	*p*	RMSEA	90% CI for RMSEA	CFI	SRMR
Structural model							
Baseline	0.000	0	0.000	0.000	[0.000 0.000]	1.000	0.000
First modification*	1.633	1	0.201	0.174	[0.000 0.638]	0.984	0.037
Second modification **	4.057	2	0.132	0.221	[0.000 0.535]	0.948	0.066

χ^2^ = Chi-Square; RMSEA = root mean square error of approximation; CI = confidence interval; CFI = comparative fit index; SRMR = standardized root mean square residual. * exclusion of the non-significant path linking Action to Social Anxiety (*p* = .192). **exclusion of the non-significant path linking Acceptance to Social Anxiety (*p* = .108)

Acceptance and action measured at T0 were negatively and directly associated with PI measured at T1. In turn, PI measured at T1 yielded a positive and direct effect on Social Anxiety measured at T2. PI totally mediated the relation between acceptance and action and social anxiety. The indirect effect of acceptance on social anxiety through PI was significant (β = −0.296, *p* < .05), as was the indirect effect of action on social anxiety through PI was also significant (β = −0.421, *p* < .001). Acceptance and action accounted for 59.6% of the variance of PI. In turn, acceptance, action, and PI collectively explained 64.2% of the social anxietys’ variance.

## Discussion

Difficulties associated with SAD usually start in adolescence (Stein et al., 2017; Knappe et al., 2015) and tend to have a chronic and unremitting course (Beesdo-Baum et al., 2012; Steinert et al., 2013), causing significant and persistent impairments in various life domains (e.g., Acquah et al., 2015; Chiu et al., 2021; Hebert et al., 2012; Soohinda & Sampath, 2016). Despite its significant prevalence rates in adolescence (e.g., Alves et al., 2022), evidence regarding the processes contributing to social anxiety in this developmental period is still scarce. There is promising evidence regarding the applicability of ACT to understand and intervene in internalizing disorders (Landy et al., 2015; Twohig & Levin, 2017), including adult (e.g., Caletti et al., 2022) and adolescent SAD (Oyetunde & Ajibola, 2019). However, evidence considering the ACT related psychological processes and their relationship with social anxiety in clinical samples of adolescents is limited. The current study aimed to bridge the existing gap on the causal role of acceptance and committed action (as PF processes) and PI on adolescents’ social anxiety over time, using an adolescent SAD sample.

As expected, our findings suggest that acceptance and committed action are negatively and directly associated with PI. This is in line with ACT’s theoretical grounds, strengthening the applicability of this model to adolescent SAD. Specifically, results support that the degree to which an adolescent with SAD is willing to be with painful internal experiences without controlling and/or trying to change them (i.e., acceptance) and is committed to acting in the presence of these difficult private events towards what is valued (i.e., committed action) has a causal and negative association with PI over time. As such, higher levels of acceptance and committed action go with lower psychological flexibility overtime (Hayes et al., 2006).

PI, subsequentially, yielded a positive and direct effect on social anxiety after 12 weeks. This is in line with previous research conducted with non-clinical adult samples (Tillfors et al., 2015) and samples of adults with anxiety disorders (Fergus et al., 2012). Our findings add to extant evidence regarding the role of PI and its processes in adolescent’s mental health difficulties, particularly SAD. As such, it seems that rigid attempts to control, alter or minimize unpleasant internal experiences, which results in an inability to change and/or persist in value-guided behaviors, leads to higher levels of social anxiety overtime in adolescents with SAD.

Additionally, PI totally mediated the relationship between acceptance and action and social anxiety. This implies that PI fully explains the association between lower levels of PF’s processes (i.e., acceptance and action) and social anxiety. Our findings suggest that, in adolescents with SAD, the willingness to experience private events without attempts to change their frequency or form (i.e., acceptance) and the ability to move towards chosen values and goals despite unpleasant internal experiences (i.e., committed action) per se do not contribute to lower levels of self-reported social anxiety overtime. Instead, though the processes negatively correlate to social anxiety, their influence is based on how they interact and contribute to PI – producing an overall rigidity and inflexibility - which, in turn, seems to contribute to the maintenance and exacerbation of social anxiety and suffering overtime. These indirect effects indicate that the tendency to be willing to be acceptant of one’s experience and to be committed with important action aligned with one’s core values may play a protective role in the development of adolescent SAD.

Overall, current findings are in line with research stating that interventions targeting PI are useful for the treatment of SAD (Caletti et al., 2022; García-Perez & Valdivia-Salas, 2018). They add to this extant literature the specificity of considering PI as an integrated process in predicting social anxiety (rather than focusing on its specific processes): focusing solely on the development/promotion of acceptance or committed action may limit the capability to reduce the heightened levels of social anxiety in adolescents. Most previous literature has, in fact, referred to PI as an integrated process, when considering its associations with social anxiety (e.g., Flynn et al., 2019). Conversely, working with the PF processes in interaction, possibly by promoting acceptance and committed action, appears to be crucial to understand and alleviate adolescents’ social anxiety. This is in line with previous research that states that interventions focusing solely on specific PF processes may limit the therapeutic potential of interventions aiming at the reduction of heightened social anxiety (Cheng et al., 2021). Alternatively, previous works consider that these processes may be better thought of as mediators of change (Forman et al., 2012), while focusing on promoting PF has resulted in beneficial outcomes for adult SAD (Caletti et al., 2022; García-Perez & Valdivia-Salas, 2018). Based on current findings, we would suggest that the same holistic and ACT-based perspective may be beneficial for adolescent SAD as well.

### Limitations

Despite the relevance of the present study, some limitations should be considered when interpreting its findings. Firstly, the current study only used self-report measures which can compromise the validity of the results. Future research should attempt to resort to alternative assessment methods and/or access the processes under study directly. Secondly, the sample size did not allow us to explore differences across samples, namely across gender and age (e.g., early and late adolescence). Considering that PI/PF processes are conceptualized as underlying overall human suffering and flourishing, with PI processes being heightened in clinical samples (e.g., cognitive fusion; Faustino et al., 2021), future studies could include clinical and non-clinical samples and explore differences between samples regarding the associations between PI/PF processes and mental health outcomes. Thirdly, not all PF processes were considered in the current work. Future research may consider the dyad theory of PF, describing PF to be organized into three pillars, each consisting of two processes (Hayes et al., 2011). As such, it seems important that future studies can evaluate the third pillar of PF that was missed in this study (e.g., contact with the present moment), as well as using measures of all PF processes, thus allowing for a more comprehensive understanding of their relationship with mental health difficulties in adolescents. Finally, it would be relevant to explore diverse directions of pathways linking our constructs of interest. We proposed that PI impacts on social anxiety over time, whereas previous works have found fear of negative evaluation, which is highly related to social anxiety, to impact on psychological inflexibility cross-sectionally (Uğur et al., 2020). It may be the case that the links between social anxiety and PI occurs in a non-recursive and self-perpetuating manner.

### Implications

The current work points to the influence of PF processes (such as acceptance and committed action) on adolescents’ social anxiety, specifically when contributing to an overall inflexible way of psychological functioning. Hence follow important theoretical and applied implications. Specifically, current evidence favors the theoretical assumption that psychological inflexibility is a dynamic process that lies at the core of human suffering over time and is supported in diverse but interrelated processes (Hayes et al., 2006, 2011); in the current work, suffering was manifested in social anxiety, and process of (lacking) acceptance and committed action were predictors of PI over time.

Moreover, though research has been promising of the efficacy of ACT for SAD (e.g., Caletti et al., 2022; García-Pérez & Valdivia-Salas, 2018), it has been focused on adult samples. The current work further sustains that ACT may be applicable to adolescent SAD, as a way to break the usually chronic and debilitating course of this disorder (e.g., Steinert et al., 2013). A qualitative approach to efficacy and contextual change would also be welcome and could potentially be achieved by using the online photovoice method. This method has allowed participants to reflect and express the facilitators and barriers for online education during COVID-19 (Doyumğaç et al., 2021). It could also be used to explore change trajectories throughout ACT interventions, thus highlighting the facilitators and barriers to change. This information may be communicated to help families and schools realize and accommodate the hardships faced by social anxious adolescents.

### Conclusions

This is one of the few studies so far that has considered the relevance of ACT related constructs to adolescent SAD, using a mediation and longitudinal approach. Findings from the current work align with previous literature on the relevance of ACT as a useful alternative to understand and change SAD, in as much as social anxiety was sustained over time by the influence of PI, in adolescents presenting with SAD. It follows that specific care should be taken to target specific ACT processes that may contribute to adolescents’ social anxiety later on, particularly at an early stage of SAD, when acceptance and action processes are still not aligned enough to directly impact social anxiety via PI. These interventions will, ultimately, contribute to buffering the lasting negative consequences of SAD and promoting adaptive adolescent life trajectories.

## Data Availability

The datasets generated during and/or analyzed during the current study are available from the corresponding author on reasonable request.

## Footnotes

1 Socioeconomic status was assessed based on Simões’ classification (1995). Low socioeconomic status include for example laborers in manufacturing; medium socioeconomic status include for example sales workers, and high socioeconomic status include for example health professionals.

## References

[CR1] Acquah EO, Topalli P, Wilson ML, Junttila N, Niemi PM (2015). Adolescent loneliness and social anxiety as predictors of bullying victimisation. International Journal of Adolescence and Youth.

[CR2] Alves F, Figueiredo DV, Vagos P (2022). The prevalence of adolescent social fears and social anxiety disorder in school contexts. International Journal of Environmental Research and Public Health.

[CR3] American Psychiatric Association (2013). *Diagnostic and statistical manual of mental disorders* (5th ed.). 10.1176/appi.books.9780890425596

[CR4] Asher M, Hofmann SG, Aderka IM (2021). I’m not feeling it: Momentary experiential avoidance and social anxiety among individuals with social anxiety disorder. Behavior Therapy.

[CR5] Bardeen JR, Fergus TA (2016). The interactive effect of cognitive fusion and experiential avoidance on anxiety, depression, stress and posttraumatic stress symptoms. Journal of Contextual Behavioral Science.

[CR6] Beesdo-Baum K, Knappe S, Fehm L, Höfler M, Lieb R, Hofmann SG, Wittchen H (2012). The natural course of social anxiety disorder among adolescents and young adults. Acta Psychiatrica Scandinavica.

[CR7] Bohlmeijer ET, Lamers SM, Fledderus M (2015). Flourishing in people with depressive symptomatology increases with acceptance and commitment therapy. Post-hoc analyses of a randomized controlled trial. Behaviour Research and Therapy.

[CR8] Caletti E, Massimo C, Magliocca S, Moltrasio C, Brambilla P, Delvecchio G (2022). The role of the acceptance and commitment therapy in the treatment of social anxiety: An updated scoping review. Journal of Affective Disorders.

[CR9] Cheng Q, Shi C, Yan C, Ren Z, Chan SH, Xiong S, Zhang T, Zheng H (2021). Sequential multiple mediation of cognitive fusion and experiential avoidance in the relationship between rumination and social anxiety among chinese adolescents. Anxiety Stress & Coping.

[CR10] Chiu K, Clark DM, Leigh E (2021). Prospective associations between peer functioning and social anxiety in adolescents: A systematic review and meta-analysis. Journal of Affective Disorders.

[CR11] Ciarrochi J, Kashdan TB, Leeson P, Heaven P, Jordan C (2010). On being aware and accepting: A one-year longitudinal study into adolescent well‐being. Journal of Adolescence.

[CR12] Cobos-Sánchez L, Flujas-Contreras JM, Becerra IG (2020). Relation between psychological flexibility, emotional intelligence and emotion regulation in adolescence. Current Psychology.

[CR13] Cookson C, Luzon O, Newland J, Kingston J (2019). Examining the role of cognitive fusion and experiential avoidance in predicting anxiety and depression. Psychology and Psychotherapy: Theory Research and Practice.

[CR14] Cunha M, Santos AM (2011). Avaliação da Inflexibilidade Psicológica em Adolescentes: Estudo das qualidades psicométricas da versão portuguesa do Avoidance and Fusion Questionnaire for Youth (AFQ-Y) [Assessment of Psychological Inflexibility in Adolescents: Study of the psychometric qualities of the portuguese version of the Avoidance and Fusion Questionnaire for Youth (AFQ-Y)]. Laboratório de Psicologia.

[CR15] Cunha M, Pinto Gouveia J, Alegre S, Salvador MC (2004). Avaliação da ansiedade na adolescência: A versão portuguesa da SAS-A [Assessment of anxiety in adolescence: The portuguese version of the SAS-A]. Psychologica.

[CR16] Cunha M, Pinto-Gouveia JP, Salvador MC (2008). Social fears in adolescence – the social anxiety and avoidance scale for adolescents. European Psychologist.

[CR17] Doyumğaç İ, Tanhan A, Kıymaz MS (2021). Understanding the most important facilitators and barriers for online education during COVID-19 through online photovoice methodology. International Journal of Higher Education.

[CR18] Faustino B (2021). Transdiagnostic perspective on psychological inflexibility and emotional dysregulation. Behavioural and Cognitive Psychotherapy.

[CR19] Faustino B, Vasco AB, Farinha-Fernandes A, Delgado J (2021). Psychological inflexibility as a transdiagnostic construct: Relationships between cognitive fusion, psychological well-being and symptomatology. Current Psychology.

[CR20] Fergus TA, Valentiner DP, Gillen MJ, Hiraoka R, Twohig MP, Abramowitz JS, McGrath PB (2012). Assessing psychological inflexibility: The psychometric properties of the avoidance and fusion questionnaire for youth in two adult samples. Psychological Assessment.

[CR21] Flynn MK, Bordieri MJ, Berkout OV (2019). Symptoms of social anxiety and depression: Acceptance of socially anxious thoughts and feelings as a moderator. Journal of Contextual Behavioral Science.

[CR22] Forman EM, Chapman JE, Herbert JD, Goetter EM, Yuen EK, Moitra E (2012). Using session-by-session measurement to compare mechanisms of action for acceptance and commitment therapy and cognitive therapy. Behavior Therapy.

[CR23] Gallego A, McHugh L, Villatte M, Lappalainen R (2020). Examining the relationship between public speaking anxiety, distress tolerance and psychological flexibility. Journal of Contextual Behavioral Science.

[CR24] García-Pérez L, Valdivia-Salas S (2018). Intervención en el trastorno de ansiedad social a través de la terapia de aceptación y compromiso: Una revisión sistemática. [Acceptance and commitment therapy for social anxiety disorder: A systematic review]. Behavioral Psychology.

[CR25] Gloster AT, Walder N, Levin M, Twohig M, Karekla M (2020). The empirical status of acceptance and commitment therapy: A review of meta-analyses. Journal of Contextual Behavioral Science.

[CR26] Greco LA, Lambert W, Baer RA (2008). Psychological inflexibility in childhood and adolescence: Development and evaluation of the Avoidance and Fusion Questionnaire for Youth. Psychological assessment.

[CR27] Hayes, S. C., & Strosahl, K. D. (Eds.). (2005). *A practical guide to acceptance and commitment therapy*. Springer Science + Business Media.

[CR28] Hayes SC, Luoma JB, Bond FW, Masuda A, Lillis J (2006). Acceptance and commitment therapy: Model, processes and outcomes. Behaviour Research and Therapy.

[CR29] Hayes, S. C., Strosahl, K. D., & Wilson, K. G. (2011). *Acceptance and Commitment Therapy, Second Edition: The process and practice of mindful change*. The Guilford Press.

[CR30] Hebert KR, Fales J, Nangle DW, Papadakis AA, Grover RL (2012). Linking social anxiety and adolescent romantic relationship functioning: Indirect effects and the importance of peers. Journal of Youth and Adolescence.

[CR31] Herbert JD, Forman EM, Kaye JL, Gershkovich M, Goetter E, Yuen EK, Glassman L, Goldstein S, Hitchcock P, Tronieri JS, Berkowitz S, Marando-Blanck S (2018). Randomized controlled trial of acceptance and commitment therapy versus traditional cognitive behavior therapy for social anxiety ddisorder: Symptomatic and behavioral outcomes. Journal of Contextual Behavioral Science.

[CR32] Hu L, Bentler PM (1999). Cutoff criteria for fit indexes in covariance structure analysis: Conventional criteria versus new alternatives. Structural Equation Modeling: A Multidisciplinary Journal.

[CR33] Jystad, I., Bjerkeset, O., Haugan, T., Sund, E. R., & Vaag, J. (2021). Sociodemographic correlates and mental health comorbidities in adolescents with social anxiety: The Young-HUNT3 study, Norway. *Frontiers in Psychology, 12*. 10.3389/fpsyg.2021.663161 10.3389/fpsyg.2021.663161 PMC8085386 33935922

[CR34] Kashdan TB, Goodman FR, Machell KA, Kleiman EM, Monfort SS, Ciarrochi J, Nezlek JB (2014). A contextual approach to experiential avoidance and social anxiety: Evidence from an experimental interaction and daily interactions of people with social anxiety disorder. Emotion.

[CR35] Kessler RC, Berglund P, Demler O, Jin R, Merikangas KR, Walters EE (2005). Lifetime prevalence and age-of-onset distributions of DSM-IV disorders in the national comorbidity survey replication. Archives of General Psychiatry.

[CR36] Khoramnia S, Bavafa A, Jaberghaderi N, Parvizifard A, Foroughi A, Ahmadi M, Amiri S (2020). The effectiveness of acceptance and commitment therapy for social anxiety disorder: A randomized clinical trial. Trends in Psychiatry and Psychotherapy.

[CR37] Kline, R. B. (2005). *Principles and practice of structural equation modeling* (2nd ed.). The Guilford Press.

[CR38] Knappe, S., Sasagawa, S., & Creswell, C. (2015). Developmental epidemiology of social anxiety and social phobia in adolescents. *Social Anxiety and Phobia in Adolescents*, 39–70. 10.1007/978-3-319-16703-9_3

[CR39] Kocovski NL, Fleming JE, Hawley LL, Huta V, Antony MM (2013). Mindfulness and acceptance-based group therapy versus traditional cognitive behavioral group therapy for social anxiety disorder: A randomized controlled trial. Behaviour Research and Therapy.

[CR40] Kocovski NL, Fleming JE, Blackie RA, MacKenzie MB, Rose AL (2019). Self-help for social anxiety: Randomized controlled trial comparing a mindfulness and acceptance-based approach with a control group. Behavior Therapy.

[CR41] La Greca AM, Lopez N (1998). Social anxiety among adolescents: Linkages with peer relations and friendships. Journal of Abnormal Child Psychology.

[CR42] Landy LN, Schneider RL, Arch JJ (2015). Acceptance and commitment therapy for the treatment of anxiety disorders: A concise review. Current Opinion in Psychology.

[CR43] Levin ME, MacLane C, Daflos S, Seeley JR, Hayes SC, Biglan A, Pistorello J (2014). Examining psychological inflexibility as a transdiagnostic process across psychological disorders. Journal of Contextual Behavioral Science.

[CR44] MacKenzie M, Kocovski N (2010). Self-reported acceptance of social anxiety symptoms: Development and validation of the social anxiety - Acceptance and Action Questionnaire. International Journal of Behavioral Consultation and Therapy.

[CR45] Makriyianis HM, Adams EA, Lozano LL, Mooney TA, Morton C, Liss M (2019). Psychological inflexibility mediates the relationship between adverse childhood experiences and mental health outcomes. Journal of Contextual Behavioral Science.

[CR46] Martins, M. J., Vieira, S., Salvador, M. C., Mackenzie, M. B., & Kocovski, M. L. (2011). Social anxiety – Acceptance and Action Questionnaire: Adaptation and validation in a Portuguese adolescent sample. Unpublished manuscript.

[CR47] McCracken LM, Badinlou F, Buhrman M, Brocki KC (2021). The role of psychological flexibility in the context of COVID-19: Associations with depression, anxiety, and insomnia. Journal of Contextual Behavioral Science.

[CR48] Merikangas KR, He JP, Burstein M, Swanson SA, Avenevoli S, Cui L, Benjet C, Georgia-des K, Swendsen J (2010). Lifetime prevalence of mental disorders in U.S. adolescents: Results from the National Comorbidity Survey replication – adolescent supplement (NCS-A). Journal of the American Academy of Child and Adolescent Psychiatry.

[CR49] Mohammadi MR, Salehi M, Khaleghi A, Hooshyari Z, Mostafavi SA, Ahmadi N, Hojjat SK, Safavi P, Amanat M (2020). Social anxiety disorder among children and adolescents: A nationwide survey of prevalence, socio-demographic characteristics, risk factors and co-morbidities. Journal of Affective Disorders.

[CR50] Muthén, L. K., & Muthén, B. O. (2010). *Mplus user’s guide* (6th ed.). Author.

[CR51] Oppo A, Schweiger M, Ristallo A, Presti G, Pergolizzi F, Moderato P (2019). Mindfulness skills and psychological inflexibility: Two useful tools for a clinical assessment for adolescents with internalizing behaviors. Journal of Child and Family Studies.

[CR52] Oyetunde S, Ajibola O (2019). Efficacy of acceptance and commitment therapy in the treatment of social phobia among adolescents in secondary schools in Oyo state, Nigeria. Annual journal of technical university of Varna Bulgaria.

[CR53] Papachristou H, Theodorou M, Neophytou K, Panayiotou G (2018). Community sample evidence on the relations among behavioural inhibition system, anxiety sensitivity, experiential avoidance, and social anxiety in adolescents. Journal of Contextual Behavioral Science.

[CR54] Räsänen P, Lappalainen P, Muotka J, Tolvanen A, Lappalainen R (2016). An online guided ACT intervention for enhancing the psychological wellbeing of university students: A randomized controlled clinical trial. Behaviour Research and Therapy.

[CR55] Rijo, D., Brazão, N., Barroso, R., Da Silva, D. R., Vagos, P., Vieira, A., Lavado, A., & Macedo, A. M. (2016). Mental health problems in male young offenders in custodial versus community based-programs: Implications for juvenile justice interventions. *Child and Adolescent Psychiatry and Mental Health*, *10*(1). 10.1186/s13034-016-0131-6 10.1186/s13034-016-0131-6 PMC5088680 27822300

[CR56] Roohi R, Soltani AA, Meimand Z, Razavi Nematollahi V (2019). The effect of Acceptance and Commitment Therapy (ACT) on increasing the self-compassion, distress tolerance, and emotion regulation in students with social anxiety disorder. Quarterly Journal of Child Mental Health.

[CR57] Salvador, M. C. (2009). *Ser eu próprio entre os outros: um novo protocolo de intervenção para adolescentes com fobia social generalizada* [*Being myself among others: A new intervention protocol for adolescents with generalized social phobia*; Unpublished doctoral dissertation]. Faculty of Psychology and Educational Sciences of University of Coimbra.

[CR58] Sheehan DV, Sheehan KH, Shytle RD, Janavs J, Bannon Y, Rogers JE, Milo KM, Stock SL, Wilkinson B (2010). Reliability and validity of the mini international neuropsychiatric interview for children and adolescents (MINI-KID). The Journal of Clinical Psychiatry.

[CR59] Shimoda Y, Ishizu K, Ohtsuki T (2018). The reciprocal relations between experiential avoidance and social anxiety among early adolescents: A prospective cohort study. Journal of Contextual Behavioral Science.

[CR60] Simões, M. M. R. (1995). *Investigações no âmbito da aferição nacional do teste das matrizes progressivas coloridas de Raven (M.P.C.R.)* [DoctoralThesis, University of Coimbra]. https://estudogeral.uc.pt/handle/10316/946

[CR61] Soohinda, G., & Sampath, H. (2016). Social phobia among school students prevalence, demographic correlates and socio-academic impairment. *Journal of Indian Association for Child and Adolescent Mental Health*, *12*(3), 211–229. 10.1177/0973134220160302

[CR62] Stein DJ, Lim C, Roest AM, de Jonge P, Aguilar-Gaxiola S, Al-Hamzawi A, Alonso J, Benjet C, Bromet EJ, Bruffaerts R, de Girolamo G, Florescu S, Gureje O, Haro JM, Harris MG, He Y, Hinkov H, Horiguchi I, Hu C, Karam A, WHO World Mental Health Survey Collaborators (2017). The cross-national epidemiology of social anxiety disorder: Data from the World Mental Health Survey Initiative. BMC medicine.

[CR63] Steinert C, Hofmann M, Leichsenring F, Kruse J (2013). What do we know today about the prospective long-term course of social anxiety disorder? A systematic literature review. Journal of Anxiety Disorders.

[CR64] Tillfors M, Toll C, Branting M, Boersma K, Jansson-Fröjmark M (2015). Allowing or fighting social anxiety: The role of psychological inflexibility in a non-clinical population. Journal of Person-Oriented Research.

[CR65] Twohig MP, Levin ME (2017). Acceptance and commitment therapy as a treatment for anxiety and depression: A review. The Psychiatric Clinics of North America.

[CR66] Uğur E, Kaya Ç, Tanhan A (2020). Psychological inflexibility mediates the relationship between fear of negative evaluation and psychological vulnerability. Current Psychology.

[CR67] Vagos P, Pereira A, Cunha M (2013). Evaluating social fears in late adolescence: Study with a portuguese sample. European Journal of Developmental Psychology.

[CR68] Venta A, Sharp C, Hart J (2012). The relation between anxiety disorder and experiential avoidance in inpatient adolescents. Psychological Assessment.

[CR69] Wersebe H, Lieb R, Meyer AH, Hofer P, Gloster AT (2018). The link between stress, well-being, and psychological flexibility during an acceptance and commitment therapy self-help intervention. International Journal of Clinical and Health Psychology.

[CR70] World Health Organization (2021, November 17). *Adolescent mental health* [Fact sheet]. Retrieved [June, 2022] from https://www.who.int/news-room/fact-sheets/detail/adolescent-mental-health

[CR71] Yabandeh MR, Bagholi H, Sarvghad S, Kouroshnia M (2019). Comparing the effectiveness of cognitive behavioral therapy with acceptance and commitment therapy on reduction of social anxiety disorder symptoms in university students. Psychological Methods and Models.

[CR72] Yadegari L, Hashemiyan K, Abolmaali K (2014). Effect of acceptance and commitment therapy on young people with social anxiety. International Journal of Scientific Research in Knowledge.

